# Mapping quantitative trait loci in line cross with repeat records

**DOI:** 10.1186/1471-2156-8-47

**Published:** 2007-07-12

**Authors:** Runqing Yang, Ming Fang

**Affiliations:** 1 School of Agriculture and Biology, Shanghai Jiaotong University, Shanghai, 201101, P.R. China; 2 Life Science College, Heilongjiang August First Land Reclamation University, Daqing, 163319, P.R. China

## Abstract

**Background:**

Phenotypes with repeat records from one individual or multiple individuals were often encountered in practices of mapping QTL in linecross. The current genetic mapping method for a trait with repeat records is adopted by simply replacing the phenotype by the average value of the repeat records. This simple treatment has not sufficiently utilized the information from the replication and ignored the impacts of the permanent environmental effects on the accuracy of the estimated QTL.

**Results:**

We propose to map QTL by using the repeatability model to directly analyze the repeat records rather than simply analyze the mean phenotype, improving the efficiency of QTL detecting because of adequately utilizing the information from data and allowing for the permanent environmental effects. A maximum likelihood method implemented via the expectation-maximization (EM) algorithm is applied to perform the parameter estimation of the repeatability model. The superiority of the mapping method based on the repeatability model over simple analysis using the mean phenotype was demonstrated by a series of simulations.

**Conclusion:**

Our results suggest that the proposed method can serve as a powerful alternative to existing methods. By mean of the repeatability model, utilizing the repeat records on individual may improve the efficiency of QTL detecting in line cross.

## Background

Replication is the fundamental of the experimental design, the important advantages of which are that it allows for an estimate of experimental error and increases the reliability of information obtained at each experimental point [1,2]. Replication denotes sampling or measuring multiple times under the same experimental condition (within one treatment), where the experimental unit may be either one individual or multiple individuals with the identical genetic background.

Often plants or animals are observed more than once for a particular trait. For examples, fleece weight of sheep in different years, blood pressure and pulse of a human over time, litter size of sows over time, antler size of deer in different seasons, racing results of horses from several races, exam scores of students during university and so on. These records observed belong to replicate ones if they are not influenced by the measuring environments, such as the years, seasons, parities, races.

In classical quantitative genetics, a trait with repeat records is generally analysed by means of the repeatability model [3,4], in which, there is an additional permanent environmental effect besides an individual's additive genetic value for a trait. The permanent environmental effect as a measure of the differences among experimental units, is a non-genetic effect common to all observations on the same individual [5]. Such environmental effects are usually accounted for in the model to ensure accurate prediction of breeding values [4]. However, the repeatability model has not been paid adequate attention to mapping QTL by using data with repeat records.

The current genetic mapping method for a trait with repeat records is adopted by simply replacing the phenotype by the average value of the repeat records [6,7]. This simple treatment has not sufficiently utilized the information from the replication and ignored the impacts of the permanent environmental effects on the accuracy of the estimated QTL, although it enables to improve the power of detecting QTL with a certain extent.

In this study, we apply the repeatability model to mapping quantitative trait loci with repeat records and demonstrate the higher efficiency of this model by the simulations.

## Theory and methods

### Mapping QTL based on the mean phenotype

Take a simple F_2 _population of size *n *derived from two homozygous lines as an example. There are the three possible genotypes denoted by *Q*_1_*Q*_1_, *Q*_1_*Q*_2_, and *Q*_2_*Q*_2_, respectively, at a quantitative trait locus *Q*. The phenotypic value of an individual *i *is usually described by the following linear model,

*y*_*i *_= *μ *+ *z*_*i*_*a *+ *w*_*i*_*d *+ *e*_*i*_,     (1)

Where *μ *is the population mean, a and d are additive and dominant effects of the QTL, *e*_*i *_is the residual error with a *N*(0, *σ*^2^) distribution, and

zi={+1for Q1Q10for Q1Q2−1for Q2Q2andwi={−1for Q1Q1+1for Q1Q2−1for Q2Q2.
 MathType@MTEF@5@5@+=feaafiart1ev1aaatCvAUfKttLearuWrP9MDH5MBPbIqV92AaeXatLxBI9gBaebbnrfifHhDYfgasaacH8akY=wiFfYdH8Gipec8Eeeu0xXdbba9frFj0=OqFfea0dXdd9vqai=hGuQ8kuc9pgc9s8qqaq=dirpe0xb9q8qiLsFr0=vr0=vr0dc8meaabaqaciaacaGaaeqabaqabeGadaaakeaafaqabeqadaaabaGaemOEaO3aaSbaaSqaaiabdMgaPbqabaGccqGH9aqpdaGabeqaauaabeqadiaaaeaacqGHRaWkcqaIXaqmaeaacqWGMbGzcqWGVbWBcqWGYbGCcqqGGaaicqqGrbqudaWgaaWcbaGaeGymaedabeaakiabbgfarnaaBaaaleaacqaIXaqmaeqaaaGcbaGaeGimaadabaGaemOzayMaem4Ba8MaemOCaiNaeeiiaaIaeeyuae1aaSbaaSqaaiabigdaXaqabaGccqqGrbqudaWgaaWcbaGaeGOmaidabeaaaOqaaiabgkHiTiabigdaXaqaaiabdAgaMjabd+gaVjabdkhaYjabbccaGiabbgfarnaaBaaaleaacqaIYaGmaeqaaOGaeeyuae1aaSbaaSqaaiabikdaYaqabaaaaaGccaGL7baaaeaacqqGHbqycqqGUbGBcqqGKbazaeaacqWG3bWDdaWgaaWcbaGaemyAaKgabeaakiabg2da9maaceqabaqbaeqabmGaaaqaaiabgkHiTiabigdaXaqaaiabdAgaMjabd+gaVjabdkhaYjabbccaGiabbgfarnaaBaaaleaacqaIXaqmaeqaaOGaeeyuae1aaSbaaSqaaiabigdaXaqabaaakeaacqGHRaWkcqaIXaqmaeaacqWGMbGzcqWGVbWBcqWGYbGCcqqGGaaicqqGrbqudaWgaaWcbaGaeGymaedabeaakiabbgfarnaaBaaaleaacqaIYaGmaeqaaaGcbaGaeyOeI0IaeGymaedabaGaemOzayMaem4Ba8MaemOCaiNaeeiiaaIaeeyuae1aaSbaaSqaaiabikdaYaqabaGccqqGrbqudaWgaaWcbaGaeGOmaidabeaaaaaakiaawUhaaaaacqGGUaGlaaa@7FA1@

If *m*_*i *_records are repeatedly sampled from each individual and the phenotypic value of an individual *i *is measured by the average of *m*_*i *_records, the model is modified as

y¯i=μ+zia+wid+e¯i,     (2)
 MathType@MTEF@5@5@+=feaafiart1ev1aaatCvAUfKttLearuWrP9MDH5MBPbIqV92AaeXatLxBI9gBaebbnrfifHhDYfgasaacH8akY=wiFfYdH8Gipec8Eeeu0xXdbba9frFj0=OqFfea0dXdd9vqai=hGuQ8kuc9pgc9s8qqaq=dirpe0xb9q8qiLsFr0=vr0=vr0dc8meaabaqaciaacaGaaeqabaqabeGadaaakeaacuWG5bqEgaqeamaaBaaaleaacqWGPbqAaeqaaOGaeyypa0dcciGae8hVd0Maey4kaSIaemOEaO3aaSbaaSqaaiabdMgaPbqabaGccqWGHbqycqGHRaWkcqWG3bWDdaWgaaWcbaGaemyAaKgabeaakiabdsgaKjabgUcaRiqbdwgaLzaaraWaaSbaaSqaaiabdMgaPbqabaGccqGGSaalaaa@41C7@

where

y¯i=1mi∑j=1miyij,e¯i=1mi∑j=1mieij,
 MathType@MTEF@5@5@+=feaafiart1ev1aaatCvAUfKttLearuWrP9MDH5MBPbIqV92AaeXatLxBI9gBaebbnrfifHhDYfgasaacH8akY=wiFfYdH8Gipec8Eeeu0xXdbba9frFj0=OqFfea0dXdd9vqai=hGuQ8kuc9pgc9s8qqaq=dirpe0xb9q8qiLsFr0=vr0=vr0dc8meaabaqaciaacaGaaeqabaqabeGadaaakeaacuWG5bqEgaqeamaaBaaaleaacqWGPbqAaeqaaOGaeyypa0ZaaSaaaeaacqaIXaqmaeaacqWGTbqBdaWgaaWcbaGaemyAaKgabeaaaaGcdaaeWbqaaiabdMha5naaBaaaleaacqWGPbqAcqWGQbGAaeqaaaqaaiabdQgaQjabg2da9iabigdaXaqaaiabd2gaTnaaBaaameaacqWGPbqAaeqaaaqdcqGHris5aOGaeiilaWIafmyzauMbaebadaWgaaWcbaGaemyAaKgabeaakiabg2da9maalaaabaGaeGymaedabaGaemyBa02aaSbaaSqaaiabdMgaPbqabaaaaOWaaabCaeaacqWGLbqzdaWgaaWcbaGaemyAaKMaemOAaOgabeaaaeaacqWGQbGAcqGH9aqpcqaIXaqmaeaacqWGTbqBdaWgaaadbaGaemyAaKgabeaaa0GaeyyeIuoakiabcYcaSaaa@5814@

and the variable with additional subscript *j *indicates the corresponding variable for the *j*th record of the *i*th F_2 _individual. The residual error now follows a *N*(0, *σ*^2^/*m*_*i*_) distribution, given that *e*_*ij *_~ *N*(0, *σ*^2^).

Let

$$f({\bar{y}}_{i}|\theta ,z_{i},w_{i})=\sqrt{\frac{m_{i}}{2\pi \sigma }}\exp\left[-\frac{m_{i}}{2\sigma^{2}}{\left({\bar{y}}_{i}-\mu -z_{i}a-w_{i}d\right)}^{2}\right]\;\left(3\right)$$

be the conditional density of y¯i
 MathType@MTEF@5@5@+=feaafiart1ev1aaatCvAUfKttLearuWrP9MDH5MBPbIqV92AaeXatLxBI9gBaebbnrfifHhDYfgasaacH8akY=wiFfYdH8Gipec8Eeeu0xXdbba9frFj0=OqFfea0dXdd9vqai=hGuQ8kuc9pgc9s8qqaq=dirpe0xb9q8qiLsFr0=vr0=vr0dc8meaabaqaciaacaGaaeqabaqabeGadaaakeaacuWG5bqEgaqeamaaBaaaleaacqWGPbqAaeqaaaaa@2FC6@, where *θ *= [*μ a d σ*^2^]^*T *^are the parameters; the log likelihood function defined under the missing variables *z*_*i *_and *w*_*i *_is

L(θ)=∑i=1nln⁡{E[f(y¯i|θ,zi,wi)]}     (4)
 MathType@MTEF@5@5@+=feaafiart1ev1aaatCvAUfKttLearuWrP9MDH5MBPbIqV92AaeXatLxBI9gBaebbnrfifHhDYfgasaacH8akY=wiFfYdH8Gipec8Eeeu0xXdbba9frFj0=OqFfea0dXdd9vqai=hGuQ8kuc9pgc9s8qqaq=dirpe0xb9q8qiLsFr0=vr0=vr0dc8meaabaqaciaacaGaaeqabaqabeGadaaakeaacqWGmbatcqGGOaakiiGacqWF4oqCcqGGPaqkcqGH9aqpdaaeWbqaaiGbcYgaSjabc6gaUbWcbaGaemyAaKMaeyypa0JaeGymaedabaGaemOBa4ganiabggHiLdGcdaGadeqaaiabdweafjabcUfaBjabdAgaMjabcIcaOiqbdMha5zaaraWaaSbaaSqaaiabdMgaPbqabaGccqGG8baFcqWF4oqCcqGGSaalcqWG6bGEdaWgaaWcbaGaemyAaKgabeaakiabcYcaSiabdEha3naaBaaaleaacqWGPbqAaeqaaOGaeiykaKIaeiyxa0facaGL7bGaayzFaaaaaa@5301@

The expectation-maximization (EM) algorithm [8] can be used to obtain the MLE, as shown below,

[∑i=1nmi∑i=1nmiE(zi)∑i=1nmiE(wi)∑i=1nmiE(zi)∑i=1nmiE(zi2)∑i=1nmiE(ziwi)∑i=1nmiE(wi)∑i=1nmiE(ziwi)∑i=1nmiE(wi2)][μad]=[∑i=1nmiy¯i∑i=1nmiE(zi)y¯i∑i=1nmiE(wi)y¯i]     (5)
 MathType@MTEF@5@5@+=feaafiart1ev1aaatCvAUfKttLearuWrP9MDH5MBPbIqV92AaeXatLxBI9gBaebbnrfifHhDYfgasaacH8akY=wiFfYdH8Gipec8Eeeu0xXdbba9frFj0=OqFfea0dXdd9vqai=hGuQ8kuc9pgc9s8qqaq=dirpe0xb9q8qiLsFr0=vr0=vr0dc8meaabaqaciaacaGaaeqabaqabeGadaaakeaadaWadaqaauaabeqadmaaaeaadaaeWaqaaiabd2gaTnaaBaaaleaacqWGPbqAaeqaaaqaaiabdMgaPjabg2da9iabigdaXaqaaiabd6gaUbqdcqGHris5aaGcbaWaaabmaeaacqWGTbqBdaWgaaWcbaGaemyAaKgabeaakiabdweafjabcIcaOiabdQha6naaBaaaleaacqWGPbqAaeqaaOGaeiykaKcaleaacqWGPbqAcqGH9aqpcqaIXaqmaeaacqWGUbGBa0GaeyyeIuoaaOqaamaaqadabaGaemyBa02aaSbaaSqaaiabdMgaPbqabaGccqWGfbqrcqGGOaakcqWG3bWDdaWgaaWcbaGaemyAaKgabeaakiabcMcaPaWcbaGaemyAaKMaeyypa0JaeGymaedabaGaemOBa4ganiabggHiLdaakeaadaaeWaqaaiabd2gaTnaaBaaaleaacqWGPbqAaeqaaOGaemyrauKaeiikaGIaemOEaO3aaSbaaSqaaiabdMgaPbqabaGccqGGPaqkaSqaaiabdMgaPjabg2da9iabigdaXaqaaiabd6gaUbqdcqGHris5aaGcbaWaaabmaeaacqWGTbqBdaWgaaWcbaGaemyAaKgabeaakiabdweafjabcIcaOiabdQha6naaDaaaleaacqWGPbqAaeaacqaIYaGmaaGccqGGPaqkaSqaaiabdMgaPjabg2da9iabigdaXaqaaiabd6gaUbqdcqGHris5aaGcbaWaaabmaeaacqWGTbqBdaWgaaWcbaGaemyAaKgabeaakiabdweafjabcIcaOiabdQha6naaBaaaleaacqWGPbqAaeqaaOGaem4DaC3aaSbaaSqaaiabdMgaPbqabaGccqGGPaqkaSqaaiabdMgaPjabg2da9iabigdaXaqaaiabd6gaUbqdcqGHris5aaGcbaWaaabmaeaacqWGTbqBdaWgaaWcbaGaemyAaKgabeaakiabdweafjabcIcaOiabdEha3naaBaaaleaacqWGPbqAaeqaaOGaeiykaKcaleaacqWGPbqAcqGH9aqpcqaIXaqmaeaacqWGUbGBa0GaeyyeIuoaaOqaamaaqadabaGaemyBa02aaSbaaSqaaiabdMgaPbqabaGccqWGfbqrcqGGOaakcqWG6bGEdaWgaaWcbaGaemyAaKgabeaakiabdEha3naaBaaaleaacqWGPbqAaeqaaOGaeiykaKcaleaacqWGPbqAcqGH9aqpcqaIXaqmaeaacqWGUbGBa0GaeyyeIuoaaOqaamaaqadabaGaemyBa02aaSbaaSqaaiabdMgaPbqabaGccqWGfbqrcqGGOaakcqWG3bWDdaqhaaWcbaGaemyAaKgabaGaeGOmaidaaOGaeiykaKcaleaacqWGPbqAcqGH9aqpcqaIXaqmaeaacqWGUbGBa0GaeyyeIuoaaaaakiaawUfacaGLDbaadaWadaqaauaabeqadeaaaeaaiiGacqWF8oqBaeaacqWGHbqyaeaacqWGKbazaaaacaGLBbGaayzxaaGaeyypa0ZaamWaaeaafaqabeWabaaabaWaaabmaeaacqWGTbqBdaWgaaWcbaGaemyAaKgabeaakiqbdMha5zaaraWaaSbaaSqaaiabdMgaPbqabaaabaGaemyAaKMaeyypa0JaeGymaedabaGaemOBa4ganiabggHiLdaakeaadaaeWaqaaiabd2gaTnaaBaaaleaacqWGPbqAaeqaaOGaemyrauKaeiikaGIaemOEaO3aaSbaaSqaaiabdMgaPbqabaGccqGGPaqkcuWG5bqEgaqeamaaBaaaleaacqWGPbqAaeqaaaqaaiabdMgaPjabg2da9iabigdaXaqaaiabd6gaUbqdcqGHris5aaGcbaWaaabmaeaacqWGTbqBdaWgaaWcbaGaemyAaKgabeaakiabdweafjabcIcaOiabdEha3naaBaaaleaacqWGPbqAaeqaaOGaeiykaKIafmyEaKNbaebadaWgaaWcbaGaemyAaKgabeaaaeaacqWGPbqAcqGH9aqpcqaIXaqmaeaacqWGUbGBa0GaeyyeIuoaaaaakiaawUfacaGLDbaaaaa@F7D2@

and

σ2=[∑i=1nmi]−1∑i=1nmiE[(y¯i−μ−zia−wid)2]     (6)
 MathType@MTEF@5@5@+=feaafiart1ev1aaatCvAUfKttLearuWrP9MDH5MBPbIqV92AaeXatLxBI9gBaebbnrfifHhDYfgasaacH8akY=wiFfYdH8Gipec8Eeeu0xXdbba9frFj0=OqFfea0dXdd9vqai=hGuQ8kuc9pgc9s8qqaq=dirpe0xb9q8qiLsFr0=vr0=vr0dc8meaabaqaciaacaGaaeqabaqabeGadaaakeaaiiGacqWFdpWCdaahaaWcbeqaaiabikdaYaaakiabg2da9maadmaabaWaaabCaeaacqWGTbqBdaWgaaWcbaGaemyAaKgabeaaaeaacqWGPbqAcqGH9aqpcqaIXaqmaeaacqWGUbGBa0GaeyyeIuoaaOGaay5waiaaw2faamaaCaaaleqabaGaeyOeI0IaeGymaedaaOWaaabCaeaacqWGTbqBdaWgaaWcbaGaemyAaKgabeaakiabdweafbWcbaGaemyAaKMaeyypa0JaeGymaedabaGaemOBa4ganiabggHiLdGcdaWadaqaaiabcIcaOiqbdMha5zaaraWaaSbaaSqaaiabdMgaPbqabaGccqGHsislcqWF8oqBcqGHsislcqWG6bGEdaWgaaWcbaGaemyAaKgabeaakiabdggaHjabgkHiTiabdEha3naaBaaaleaacqWGPbqAaeqaaOGaemizaqMaeiykaKYaaWbaaSqabeaacqaIYaGmaaaakiaawUfacaGLDbaaaaa@5EB0@

The expectation shown in Equation 6 can be further expressed as

E[y¯i−μ−zia−wid)2]=(y¯i−μ)2+a2E(zi2)+d2E(wi2)−2(y¯i−μ)[aE(zi)+dE(wi)]+2adE(ziwi)
 MathType@MTEF@5@5@+=feaafiart1ev1aaatCvAUfKttLearuWrP9MDH5MBPbIqV92AaeXatLxBI9gBaebbnrfifHhDYfgasaacH8akY=wiFfYdH8Gipec8Eeeu0xXdbba9frFj0=OqFfea0dXdd9vqai=hGuQ8kuc9pgc9s8qqaq=dirpe0xb9q8qiLsFr0=vr0=vr0dc8meaabaqaciaacaGaaeqabaqabeGadaaakeaafaqaaeGabaaabaGaemyrauKaei4waSLafmyEaKNbaebadaWgaaWcbaGaemyAaKgabeaakiabgkHiTGGaciab=X7aTjabgkHiTiabdQha6naaBaaaleaacqWGPbqAaeqaaOGaemyyaeMaeyOeI0Iaem4DaC3aaSbaaSqaaiabdMgaPbqabaGccqWGKbazcqGGPaqkdaahaaWcbeqaaiabikdaYaaakiabc2faDbqacmaaWdaaOfaamgGaaCzcaiabg2da9iabcIcaOiqbdMha5zaaraWaaSbaaSqaaiabdMgaPbqabaGccqGHsislcqWF8oqBcqGGPaqkdaahaaWcbeqaaiabikdaYaaakiabgUcaRiabdggaHnaaCaaaleqabaGaeGOmaidaaOGaemyrauKaeiikaGIaemOEaO3aa0baaSqaaiabdMgaPbqaaiabikdaYaaakiabcMcaPiabgUcaRiabdsgaKnaaCaaaleqabaGaeGOmaidaaOGaemyrauKaeiikaGIaem4DaC3aa0baaSqaaiabdMgaPbqaaiabikdaYaaakiabcMcaPiabgkHiTiabikdaYiabcIcaOiqbdMha5zaaraWaaSbaaSqaaiabdMgaPbqabaGccqGHsislcqWF8oqBcqGGPaqkcqGGBbWwcqWGHbqycqWGfbqrcqGGOaakcqWG6bGEdaWgaaWcbaGaemyAaKgabeaakiabcMcaPiabgUcaRiabdsgaKjabdweafjabcIcaOiabdEha3naaBaaaleaacqWGPbqAaeqaaOGaeiykaKIaeiyxa0Laey4kaSIaeGOmaiJaemyyaeMaemizaqMaemyrauKaeiikaGIaemOEaO3aaSbaaSqaaiabdMgaPbqabaGccqWG3bWDdaWgaaWcbaGaemyAaKgabeaakiabcMcaPaaaaaa@8B4C@

Define the posterior probabilities of the three QTL genotypes for *j*th individual as

pik∗=pikf(y¯i|θ,zi,wi)∑k=13pikf(y¯i|θ,zi,wi)(k=1,2,3),     (7)
 MathType@MTEF@5@5@+=feaafiart1ev1aaatCvAUfKttLearuWrP9MDH5MBPbIqV92AaeXatLxBI9gBaebbnrfifHhDYfgasaacH8akY=wiFfYdH8Gipec8Eeeu0xXdbba9frFj0=OqFfea0dXdd9vqai=hGuQ8kuc9pgc9s8qqaq=dirpe0xb9q8qiLsFr0=vr0=vr0dc8meaabaqaciaacaGaaeqabaqabeGadaaakeaafaqabeqacaaabaGaemiCaa3aa0baaSqaaiabdMgaPjabdUgaRbqaaiabgEHiQaaakiabg2da9maalaaabaGaemiCaa3aaSbaaSqaaiabdMgaPjabdUgaRbqabaGccqWGMbGzcqGGOaakcuWG5bqEgaqeamaaBaaaleaacqWGPbqAaeqaaOGaeiiFaWhcciGae8hUdeNaeiilaWIaemOEaO3aaSbaaSqaaiabdMgaPbqabaGccqGGSaalcqWG3bWDdaWgaaWcbaGaemyAaKgabeaakiabcMcaPaqaamaaqadabaGaemiCaa3aaSbaaSqaaiabdMgaPjabdUgaRbqabaaabaGaem4AaSMaeyypa0JaeGymaedabaGaeG4mamdaniabggHiLdGccqWGMbGzcqGGOaakcuWG5bqEgaqeamaaBaaaleaacqWGPbqAaeqaaOGaeiiFaWNae8hUdeNaeiilaWIaemOEaO3aaSbaaSqaaiabdMgaPbqabaGccqGGSaalcqWG3bWDdaWgaaWcbaGaemyAaKgabeaakiabcMcaPaaaaeaacqGGOaakcqWGRbWAcqGH9aqpcqaIXaqmcqGGSaalcqaIYaGmcqGGSaalcqaIZaWmcqGGPaqkcqGGSaalaaaaaa@6E07@

where *p*_*ik *_are the conditional probabilities inferred by marker information, then

E(zi)=pi1∗−pi3∗,E(zi2)=pi1∗+pi3∗,E(wi)=pi2∗−pi1∗−pi3∗,E(wi2)=1 and E(ziwi)=pi3∗−pi1∗.     (8)
 MathType@MTEF@5@5@+=feaafiart1ev1aaatCvAUfKttLearuWrP9MDH5MBPbIqV92AaeXatLxBI9gBaebbnrfifHhDYfgasaacH8akY=wiFfYdH8Gipec8Eeeu0xXdbba9frFj0=OqFfea0dXdd9vqai=hGuQ8kuc9pgc9s8qqaq=dirpe0xb9q8qiLsFr0=vr0=vr0dc8meaabaqaciaacaGaaeqabaqabeGadaaakeaafaqaaeGabaaabaGaemyrauKaeiikaGIaemOEaO3aaSbaaSqaaiabdMgaPbqabaGccqGGPaqkcqGH9aqpcqWGWbaCdaqhaaWcbaGaemyAaKMaeGymaedabaGaey4fIOcaaOGaeyOeI0IaemiCaa3aa0baaSqaaiabdMgaPjabiodaZaqaaiabgEHiQaaakiabcYcaSiabdweafjabcIcaOiabdQha6naaDaaaleaacqWGPbqAaeaacqaIYaGmaaGccqGGPaqkcqGH9aqpcqWGWbaCdaqhaaWcbaGaemyAaKMaeGymaedabaGaey4fIOcaaOGaey4kaSIaemiCaa3aa0baaSqaaiabdMgaPjabiodaZaqaaiabgEHiQaaakiabcYcaSaqaaiabdweafjabcIcaOiabdEha3naaBaaaleaacqWGPbqAaeqaaOGaeiykaKIaeyypa0JaemiCaa3aa0baaSqaaiabdMgaPjabikdaYaqaaiabgEHiQaaakiabgkHiTiabdchaWnaaDaaaleaacqWGPbqAcqaIXaqmaeaacqGHxiIkaaGccqGHsislcqWGWbaCdaqhaaWcbaGaemyAaKMaeG4mamdabaGaey4fIOcaaOGaeiilaWIaemyrauKaeiikaGIaem4DaC3aa0baaSqaaiabdMgaPbqaaiabikdaYaaakiabcMcaPiabg2da9iabigdaXiabbccaGiabbggaHjabb6gaUjabbsgaKjabbccaGiabdweafjabcIcaOiabdQha6naaBaaaleaacqWGPbqAaeqaaOGaem4DaC3aaSbaaSqaaiabdMgaPbqabaGccqGGPaqkcqGH9aqpcqWGWbaCdaqhaaWcbaGaemyAaKMaeG4mamdabaGaey4fIOcaaOGaeyOeI0IaemiCaa3aa0baaSqaaiabdMgaPjabigdaXaqaaiabgEHiQaaakiabc6caUaaaaaa@8E26@

Because pik∗
 MathType@MTEF@5@5@+=feaafiart1ev1aaatCvAUfKttLearuWrP9MDH5MBPbIqV92AaeXatLxBI9gBaebbnrfifHhDYfgasaacH8akY=wiFfYdH8Gipec8Eeeu0xXdbba9frFj0=OqFfea0dXdd9vqai=hGuQ8kuc9pgc9s8qqaq=dirpe0xb9q8qiLsFr0=vr0=vr0dc8meaabaqaciaacaGaaeqabaqabeGadaaakeaacqWGWbaCdaqhaaWcbaGaemyAaKMaem4AaSgabaGaey4fIOcaaaaa@31EB@ is a function of the unknown parameters, iterations are required for EM algorithm. The iterations are described as

*Step 0*: Set up initials for *θ*^(0)^.

*Step 1*: Calculate the posterior probabilities pik∗
 MathType@MTEF@5@5@+=feaafiart1ev1aaatCvAUfKttLearuWrP9MDH5MBPbIqV92AaeXatLxBI9gBaebbnrfifHhDYfgasaacH8akY=wiFfYdH8Gipec8Eeeu0xXdbba9frFj0=OqFfea0dXdd9vqai=hGuQ8kuc9pgc9s8qqaq=dirpe0xb9q8qiLsFr0=vr0=vr0dc8meaabaqaciaacaGaaeqabaqabeGadaaakeaacqWGWbaCdaqhaaWcbaGaemyAaKMaem4AaSgabaGaey4fIOcaaaaa@31EB@ with equation (7).

*Step 2*: Substituting (8) into equation (5), estimate

μ(1)=[∑i=1nmi]−1[∑i=1nmiy¯i−a(0)∑i=1nmi(pi1∗−pi3∗)−d(0)∑i=1nmi(pi2∗−pi1∗−pi3∗)]a(1)=[∑i=1nmi(pi1∗+pi3∗)]−1[∑i=1nmi(pi1∗−pi3∗)y¯i−μ(1)∑i=1nmi(pi1∗−pi3∗)−d(0)∑i=1nmi(pi3∗−pi1∗)]d(1)=[∑i=1nmi]−1[∑i=1nmi(pi2∗−pi1∗−pi3∗)y¯i−μ(1)∑i=1nmi(pi2∗−pi1∗−pi3∗)−a(1)∑i=1nmi(pi3∗−pi1∗)]     (9)
 MathType@MTEF@5@5@+=feaafiart1ev1aaatCvAUfKttLearuWrP9MDH5MBPbIqV92AaeXatLxBI9gBaebbnrfifHhDYfgasaacH8akY=wiFfYdH8Gipec8Eeeu0xXdbba9frFj0=OqFfea0dXdd9vqai=hGuQ8kuc9pgc9s8qqaq=dirpe0xb9q8qiLsFr0=vr0=vr0dc8meaabaqaciaacaGaaeqabaqabeGadaaakeaafaqaaeWabaaabaacciGae8hVd02aaWbaaSqabeaacqGGOaakcqaIXaqmcqGGPaqkaaGccqGH9aqpdaWadaqaamaaqadabaGaemyBa02aaSbaaSqaaiabdMgaPbqabaaabaGaemyAaKMaeyypa0JaeGymaedabaGaemOBa4ganiabggHiLdaakiaawUfacaGLDbaadaahaaWcbeqaaiabgkHiTiabigdaXaaakmaadmaabaWaaabmaeaacqWGTbqBdaWgaaWcbaGaemyAaKgabeaakiqbdMha5zaaraWaaSbaaSqaaiabdMgaPbqabaaabaGaemyAaKMaeyypa0JaeGymaedabaGaemOBa4ganiabggHiLdGccqGHsislcqWGHbqydaahaaWcbeqaaiabcIcaOiabicdaWiabcMcaPaaakmaaqadabaGaemyBa02aaSbaaSqaaiabdMgaPbqabaGccqGGOaakcqWGWbaCdaqhaaWcbaGaemyAaKMaeGymaedabaGaey4fIOcaaOGaeyOeI0IaemiCaa3aa0baaSqaaiabdMgaPjabiodaZaqaaiabgEHiQaaakiabcMcaPiabgkHiTiabdsgaKnaaCaaaleqabaGaeiikaGIaeGimaaJaeiykaKcaaOWaaabmaeaacqWGTbqBdaWgaaWcbaGaemyAaKgabeaaaeaacqWGPbqAcqGH9aqpcqaIXaqmaeaacqWGUbGBa0GaeyyeIuoakiabcIcaOiabdchaWnaaDaaaleaacqWGPbqAcqaIYaGmaeaacqGHxiIkaaGccqGHsislcqWGWbaCdaqhaaWcbaGaemyAaKMaeGymaedabaGaey4fIOcaaOGaeyOeI0IaemiCaa3aa0baaSqaaiabdMgaPjabiodaZaqaaiabgEHiQaaakiabcMcaPaWcbaGaemyAaKMaeyypa0JaeGymaedabaGaemOBa4ganiabggHiLdaakiaawUfacaGLDbaaaeaacqWGHbqydaahaaWcbeqaaiabcIcaOiabigdaXiabcMcaPaaakiabg2da9maadmaabaWaaabmaeaacqWGTbqBdaWgaaWcbaGaemyAaKgabeaaaeaacqWGPbqAcqGH9aqpcqaIXaqmaeaacqWGUbGBa0GaeyyeIuoakiabcIcaOiabdchaWnaaDaaaleaacqWGPbqAcqaIXaqmaeaacqGHxiIkaaGccqGHRaWkcqWGWbaCdaqhaaWcbaGaemyAaKMaeG4mamdabaGaey4fIOcaaOGaeiykaKcacaGLBbGaayzxaaWaaWbaaSqabeaacqGHsislcqaIXaqmaaGcdaWadaqaamaaqadabaGaemyBa02aaSbaaSqaaiabdMgaPbqabaGccqGGOaakcqWGWbaCdaqhaaWcbaGaemyAaKMaeGymaedabaGaey4fIOcaaOGaeyOeI0IaemiCaa3aa0baaSqaaiabdMgaPjabiodaZaqaaiabgEHiQaaakiabcMcaPiqbdMha5zaaraWaaSbaaSqaaiabdMgaPbqabaaabaGaemyAaKMaeyypa0JaeGymaedabaGaemOBa4ganiabggHiLdGccqGHsislcqWF8oqBdaahaaWcbeqaaiabcIcaOiabigdaXiabcMcaPaaakmaaqadabaGaemyBa02aaSbaaSqaaiabdMgaPbqabaGccqGGOaakcqWGWbaCdaqhaaWcbaGaemyAaKMaeGymaedabaGaey4fIOcaaOGaeyOeI0IaemiCaa3aa0baaSqaaiabdMgaPjabiodaZaqaaiabgEHiQaaakiabcMcaPiabgkHiTiabdsgaKnaaCaaaleqabaGaeiikaGIaeGimaaJaeiykaKcaaOWaaabmaeaacqWGTbqBdaWgaaWcbaGaemyAaKgabeaaaeaacqWGPbqAcqGH9aqpcqaIXaqmaeaacqWGUbGBa0GaeyyeIuoakiabcIcaOiabdchaWnaaDaaaleaacqWGPbqAcqaIZaWmaeaacqGHxiIkaaGccqGHsislcqWGWbaCdaqhaaWcbaGaemyAaKMaeGymaedabaGaey4fIOcaaOGaeiykaKcaleaacqWGPbqAcqGH9aqpcqaIXaqmaeaacqWGUbGBa0GaeyyeIuoaaOGaay5waiaaw2faaaqaaiabdsgaKnaaCaaaleqabaGaeiikaGIaeGymaeJaeiykaKcaaOGaeyypa0ZaamWaaeaadaaeWaqaaiabd2gaTnaaBaaaleaacqWGPbqAaeqaaaqaaiabdMgaPjabg2da9iabigdaXaqaaiabd6gaUbqdcqGHris5aaGccaGLBbGaayzxaaWaaWbaaSqabeaacqGHsislcqaIXaqmaaGcdaWadaqaamaaqadabaGaemyBa02aaSbaaSqaaiabdMgaPbqabaGccqGGOaakcqWGWbaCdaqhaaWcbaGaemyAaKMaeGOmaidabaGaey4fIOcaaOGaeyOeI0IaemiCaa3aa0baaSqaaiabdMgaPjabigdaXaqaaiabgEHiQaaakiabgkHiTiabdchaWnaaDaaaleaacqWGPbqAcqaIZaWmaeaacqGHxiIkaaGccqGGPaqkcuWG5bqEgaqeamaaBaaaleaacqWGPbqAaeqaaaqaaiabdMgaPjabg2da9iabigdaXaqaaiabd6gaUbqdcqGHris5aOGaeyOeI0Iae8hVd02aaWbaaSqabeaacqGGOaakcqaIXaqmcqGGPaqkaaGcdaaeWaqaaiabd2gaTnaaBaaaleaacqWGPbqAaeqaaOGaeiikaGIaemiCaa3aa0baaSqaaiabdMgaPjabikdaYaqaaiabgEHiQaaakiabgkHiTiabdchaWnaaDaaaleaacqWGPbqAcqaIXaqmaeaacqGHxiIkaaGccqGHsislcqWGWbaCdaqhaaWcbaGaemyAaKMaeG4mamdabaGaey4fIOcaaOGaeiykaKIaeyOeI0Iaemyyae2aaWbaaSqabeaacqGGOaakcqaIXaqmcqGGPaqkaaGcdaaeWaqaaiabd2gaTnaaBaaaleaacqWGPbqAaeqaaaqaaiabdMgaPjabg2da9iabigdaXaqaaiabd6gaUbqdcqGHris5aOGaeiikaGIaemiCaa3aa0baaSqaaiabdMgaPjabiodaZaqaaiabgEHiQaaakiabgkHiTiabdchaWnaaDaaaleaacqWGPbqAcqaIXaqmaeaacqGHxiIkaaGccqGGPaqkaSqaaiabdMgaPjabg2da9iabigdaXaqaaiabd6gaUbqdcqGHris5aaGccaGLBbGaayzxaaaaaaaa@6B66@

*Step 3*: Substituting (8) into equation (6), estimate

σ2(1)=[∑i=1nmi]−1{∑i=1nmi[pi1∗(y¯i−μ(1)−a(1)+d(1))2+pi2∗(y¯i−μ(1)−d(1))2+pi3∗(y¯i−μ(1)+a(1)+d(1))2]}
 MathType@MTEF@5@5@+=feaafiart1ev1aaatCvAUfKttLearuWrP9MDH5MBPbIqV92AaeXatLxBI9gBaebbnrfifHhDYfgasaacH8akY=wiFfYdH8Gipec8Eeeu0xXdbba9frFj0=OqFfea0dXdd9vqai=hGuQ8kuc9pgc9s8qqaq=dirpe0xb9q8qiLsFr0=vr0=vr0dc8meaabaqaciaacaGaaeqabaqabeGadaaakeaaiiGacqWFdpWCdaahaaWcbeqaaiabikdaYiabcIcaOiabigdaXiabcMcaPaaakiabg2da9maadmaabaWaaabCaeaacqWGTbqBdaWgaaWcbaGaemyAaKgabeaaaeaacqWGPbqAcqGH9aqpcqaIXaqmaeaacqWGUbGBa0GaeyyeIuoaaOGaay5waiaaw2faamaaCaaaleqabaGaeyOeI0IaeGymaedaaOWaaiWabeaadaaeWbqaaiabd2gaTnaaBaaaleaacqWGPbqAaeqaaOWaamWaaeaacqWGWbaCdaqhaaWcbaGaemyAaKMaeGymaedabaGaey4fIOcaaOWaaeWaaeaacuWG5bqEgaqeamaaBaaaleaacqWGPbqAaeqaaOGaeyOeI0IamGjG=X7aTnaaCaaaleqabaGaeiikaGIaeGymaeJaeiykaKcaaOGaeyOeI0Iaemyyae2aaWbaaSqabeaacqGGOaakcqaIXaqmcqGGPaqkaaGccqGHRaWkcqWGKbazdaahaaWcbeqaaiabcIcaOiabigdaXiabcMcaPaaaaOGaayjkaiaawMcaamaaCaaaleqabaGaeGOmaidaaOGaey4kaSIaemiCaa3aa0baaSqaaiabdMgaPjabikdaYaqaaiabgEHiQaaakmaabmaabaGafmyEaKNbaebadaWgaaWcbaGaemyAaKgabeaakiabgkHiTiab=X7aTnaaCaaaleqabaGaeiikaGIaeGymaeJaeiykaKcaaOGaeyOeI0Iaemizaq2aaWbaaSqabeaacqGGOaakcqaIXaqmcqGGPaqkaaaakiaawIcacaGLPaaadaahaaWcbeqaaiabikdaYaaakiabgUcaRiabdchaWnaaDaaaleaacqWGPbqAcqaIZaWmaeaacqGHxiIkaaGcdaqadaqaaiqbdMha5zaaraWaaSbaaSqaaiabdMgaPbqabaGccqGHsislcqWF8oqBdaahaaWcbeqaaiabcIcaOiabigdaXiabcMcaPaaakiabgUcaRiabdggaHnaaCaaaleqabaGaeiikaGIaeGymaeJaeiykaKcaaOGaey4kaSIaemizaq2aaWbaaSqabeaacqGGOaakcqaIXaqmcqGGPaqkaaaakiaawIcacaGLPaaadaahaaWcbeqaaiabikdaYaaaaOGaay5waiaaw2faaaWcbaGaemyAaKMaeyypa0JaeGymaedabaGaemOBa4ganiabggHiLdaakiaawUhacaGL9baaaaa@9BED@

*Step 4*: Go to step 1, which complete one round of iteration.

### Mapping QTL based on the repeatability model

Partitioning residual error *e*_*i *_in model (1) into an individual-specific permanent environmental effect *ζ*_*i *_and random environmental effect *ε*_*ij*_, the *j*th phenotypic value of an individual *i *is represented as

*y*_*ij *_= *μ *+ *z*_*i*_*a *+ *w*_*i*_*d *+ *ζ*_*i *_+ *ε *_*ij*_     (10)

This is a mixed effects model, also called repeatability model, with *a *and *d *being treated as the fixed effects and *p*_*i *_as the random effect. i.i.d. *N*(0, σζ2
 MathType@MTEF@5@5@+=feaafiart1ev1aaatCvAUfKttLearuWrP9MDH5MBPbIqV92AaeXatLxBI9gBaebbnrfifHhDYfgasaacH8akY=wiFfYdH8Gipec8Eeeu0xXdbba9frFj0=OqFfea0dXdd9vqai=hGuQ8kuc9pgc9s8qqaq=dirpe0xb9q8qiLsFr0=vr0=vr0dc8meaabaqaciaacaGaaeqabaqabeGadaaakeaaiiGacqWFdpWCdaqhaaWcbaGae8NTdOhabaGaeGOmaidaaaaa@314D@) distribution and *ε *_*ij *_i.i.d. *N*(0, σε2
 MathType@MTEF@5@5@+=feaafiart1ev1aaatCvAUfKttLearuWrP9MDH5MBPbIqV92AaeXatLxBI9gBaebbnrfifHhDYfgasaacH8akY=wiFfYdH8Gipec8Eeeu0xXdbba9frFj0=OqFfea0dXdd9vqai=hGuQ8kuc9pgc9s8qqaq=dirpe0xb9q8qiLsFr0=vr0=vr0dc8meaabaqaciaacaGaaeqabaqabeGadaaakeaaiiGacqWFdpWCdaqhaaWcbaGae8xTdugabaGaeGOmaidaaaaa@3137@) distribution.

We use an *m*_*i *_× 1 vector *y*_*i *_= [*y*_*i*1 _*y*_*i*2 _… *y*_*im *_]^*T*^, for *n *= 1, 2, …, *n *to denote the array of phenotypic values for the *i*th individual and define *ϕ*_*i *_= [1 1 … 1]^*T *^as a vector of dimension *m*_*i*_. In matrix notation, model (9) can be written as

*y*_*i *_= *ϕ*_*i*_*μ *+ *z*_*i*_*ϕ*_*i*_*a *+ *w*_*i*_*ϕ*_*i*_*d *+ *ϕ*_*i*_*ζ*_*i *_+ *ε*_*i*_     (11)

where *ε*_*i *_= [*ε*_*i*1 _*ε*_*i*2 _* … ε*_*im*_]^*T *^is an *m*_*i*_*× *1 vector for the random environmental effects which follows *N*(0, *I*_*i*_, σε2
 MathType@MTEF@5@5@+=feaafiart1ev1aaatCvAUfKttLearuWrP9MDH5MBPbIqV92AaeXatLxBI9gBaebbnrfifHhDYfgasaacH8akY=wiFfYdH8Gipec8Eeeu0xXdbba9frFj0=OqFfea0dXdd9vqai=hGuQ8kuc9pgc9s8qqaq=dirpe0xb9q8qiLsFr0=vr0=vr0dc8meaabaqaciaacaGaaeqabaqabeGadaaakeaaiiGacqWFdpWCdaqhaaWcbaGae8xTdugabaGaeGOmaidaaaaa@3137@) with *I*_*i *_being an (*m*_*i*_*× *1) × (*m*_*i*_*× *1) identity matrix. The conditional expectation of model (11) given the fixed effects is

*E*(*y*_*i*_) = *M*_*i *_= *ϕ*_*i*_*μ *+ *z*_*i*_*ϕ*_*i*_*a *+ *w*_*i*_*ϕ*_*i*_*d*     (12)

and the variance-covariance matrix is

Var(yi)=Vi=φiφiTσζ2+Iiσε2     (13)
 MathType@MTEF@5@5@+=feaafiart1ev1aaatCvAUfKttLearuWrP9MDH5MBPbIqV92AaeXatLxBI9gBaebbnrfifHhDYfgasaacH8akY=wiFfYdH8Gipec8Eeeu0xXdbba9frFj0=OqFfea0dXdd9vqai=hGuQ8kuc9pgc9s8qqaq=dirpe0xb9q8qiLsFr0=vr0=vr0dc8meaabaqaciaacaGaaeqabaqabeGadaaakeaacqWGwbGvcqWGHbqycqWGYbGCcqGGOaakcqWG5bqEdaWgaaWcbaGaemyAaKgabeaakiabcMcaPiabg2da9iabdAfawnaaBaaaleaacqWGPbqAaeqaaOGaeyypa0dcciGae8NXdy2aaSbaaSqaaiabdMgaPbqabaGccqWFgpGzdaqhaaWcbaGaemyAaKgabaGaemivaqfaaOGae83Wdm3aa0baaSqaaiab=z7a6bqaaiabikdaYaaakiabgUcaRiabdMeajnaaBaaaleaacqWGPbqAaeqaaOGae83Wdm3aa0baaSqaaiab=v7aLbqaaiabikdaYaaaaaa@4E9D@

which applies to all *i *= 1, 2, …, *n*.

The conditional density of *y*_*i *_based on *M*_*i *_and *V*_*i *_is

f(yi|θ,zi,wi)=12π|Vi|1/2exp⁡[−12(yi−Mi)TVi−1(yi−Mi)]     (14)
 MathType@MTEF@5@5@+=feaafiart1ev1aaatCvAUfKttLearuWrP9MDH5MBPbIqV92AaeXatLxBI9gBaebbnrfifHhDYfgasaacH8akY=wiFfYdH8Gipec8Eeeu0xXdbba9frFj0=OqFfea0dXdd9vqai=hGuQ8kuc9pgc9s8qqaq=dirpe0xb9q8qiLsFr0=vr0=vr0dc8meaabaqaciaacaGaaeqabaqabeGadaaakeaacqWGMbGzcqGGOaakcqWG5bqEdaWgaaWcbaGaemyAaKgabeaakiabcYha8HGaciab=H7aXjabcYcaSiabdQha6naaBaaaleaacqWGPbqAaeqaaOGaeiilaWIaem4DaC3aaSbaaSqaaiabdMgaPbqabaGccqGGPaqkcqGH9aqpdaWcaaqaaiabigdaXaqaamaakaaabaGaeGOmaiJae8hWdahaleqaaOWaaqWaaeaacqWGwbGvdaWgaaWcbaGaemyAaKgabeaaaOGaay5bSlaawIa7amaaCaaaleqabaGaeGymaeJaei4la8IaeGOmaidaaaaakiGbcwgaLjabcIha4jabcchaWnaadmaabaGaeyOeI0YaaSaaaeaacqaIXaqmaeaacqaIYaGmaaGaeiikaGIaemyEaK3aaSbaaSqaaiabdMgaPbqabaGccqGHsislcqWGnbqtdaWgaaWcbaGaemyAaKgabeaakiabcMcaPmaaCaaaleqabaGaemivaqfaaOGaemOvay1aa0baaSqaaiabdMgaPbqaaiabgkHiTiabigdaXaaakiabcIcaOiabdMha5naaBaaaleaacqWGPbqAaeqaaOGaeyOeI0Iaemyta00aaSbaaSqaaiabdMgaPbqabaGccqGGPaqkaiaawUfacaGLDbaaaaa@6B5D@

where *θ *= [*μ a d *σp2
 MathType@MTEF@5@5@+=feaafiart1ev1aaatCvAUfKttLearuWrP9MDH5MBPbIqV92AaeXatLxBI9gBaebbnrfifHhDYfgasaacH8akY=wiFfYdH8Gipec8Eeeu0xXdbba9frFj0=OqFfea0dXdd9vqai=hGuQ8kuc9pgc9s8qqaq=dirpe0xb9q8qiLsFr0=vr0=vr0dc8meaabaqaciaacaGaaeqabaqabeGadaaakeaaiiGacqWFdpWCdaqhaaWcbaGaemiCaahabaGaeGOmaidaaaaa@30FE@σε2
 MathType@MTEF@5@5@+=feaafiart1ev1aaatCvAUfKttLearuWrP9MDH5MBPbIqV92AaeXatLxBI9gBaebbnrfifHhDYfgasaacH8akY=wiFfYdH8Gipec8Eeeu0xXdbba9frFj0=OqFfea0dXdd9vqai=hGuQ8kuc9pgc9s8qqaq=dirpe0xb9q8qiLsFr0=vr0=vr0dc8meaabaqaciaacaGaaeqabaqabeGadaaakeaaiiGacqWFdpWCdaqhaaWcbaGae8xTdugabaGaeGOmaidaaaaa@3137@]. Corresponding log-likelihood function defined is

L(θ)=∑i=1nln⁡{E[f(yi|θ,zi,wi)]}     (15)
 MathType@MTEF@5@5@+=feaafiart1ev1aaatCvAUfKttLearuWrP9MDH5MBPbIqV92AaeXatLxBI9gBaebbnrfifHhDYfgasaacH8akY=wiFfYdH8Gipec8Eeeu0xXdbba9frFj0=OqFfea0dXdd9vqai=hGuQ8kuc9pgc9s8qqaq=dirpe0xb9q8qiLsFr0=vr0=vr0dc8meaabaqaciaacaGaaeqabaqabeGadaaakeaacqWGmbatcqGGOaakiiGacqWF4oqCcqGGPaqkcqGH9aqpdaaeWbqaaiGbcYgaSjabc6gaUbWcbaGaemyAaKMaeyypa0JaeGymaedabaGaemOBa4ganiabggHiLdGcdaGadeqaaiabdweafjabcUfaBjabdAgaMjabcIcaOiabdMha5naaBaaaleaacqWGPbqAaeqaaOGaeiiFaWNae8hUdeNaeiilaWIaemOEaO3aaSbaaSqaaiabdMgaPbqabaGccqGGSaalcqWG3bWDdaWgaaWcbaGaemyAaKgabeaakiabcMcaPiabc2faDbGaay5Eaiaaw2haaaaa@52E9@

With derivative for *μ*, a and d, we can obtain

$$\left[\begin{array}{ccc} \sum_{i-1}^{n}\varphi_{i}^{T}V_{i}^{-1}\varphi_{i} & \sum_{i=1}^{n}E(z_{i})\varphi_{i}^{T}V_{i}^{-1}\varphi_{i} & \sum_{i=1}^{n}E(w_{i})\varphi_{i}^{T}V_{i}^{-1}\varphi_{i}\\ \sum_{i=1}^{n}E(z_{i})\varphi_{i}^{T}V_{i}^{-1}\varphi_{i} & \sum_{i=1}^{n}E(z_{i}^{2})\varphi_{i}^{T}V_{i}^{-1}\varphi_{i} & \sum_{i=1}^{n}E(z_{i}w_{i})\varphi_{i}^{T}V_{i}^{-1}\varphi_{i}\\ \sum_{i=1}^{n}E(w_{i})\varphi_{i}^{T}V_{i}^{-1}\varphi_{i} & \sum_{i=1}^{n}E(z_{i}w_{i})\varphi_{i}^{T}V_{i}^{-1}\varphi_{i} & \sum_{i=1}^{n}E(w_{i}^{2})\varphi_{i}^{T}V_{i}^{-1}\varphi_{i} \end{array}\right]\left[\begin{array}{c} \mu \\ a\\ d \end{array}\right]=\left[\begin{array}{c} \sum_{i=1}^{n}\varphi_{i}^{T}V_{i}^{-1}y_{i}\\ \sum_{i=1}^{n}E(z_{i})\varphi_{i}^{T}V_{i}^{-1}y_{i}\\ \sum_{i=1}^{n}E(w_{i})\varphi_{i}^{T}V_{i}^{-1}y_{i} \end{array}\right],\;\left(16\right)$$

but the explicit equations for σζ2
 MathType@MTEF@5@5@+=feaafiart1ev1aaatCvAUfKttLearuWrP9MDH5MBPbIqV92AaeXatLxBI9gBaebbnrfifHhDYfgasaacH8akY=wiFfYdH8Gipec8Eeeu0xXdbba9frFj0=OqFfea0dXdd9vqai=hGuQ8kuc9pgc9s8qqaq=dirpe0xb9q8qiLsFr0=vr0=vr0dc8meaabaqaciaacaGaaeqabaqabeGadaaakeaaiiGacqWFdpWCdaqhaaWcbaGae8NTdOhabaGaeGOmaidaaaaa@314D@ and σε2
 MathType@MTEF@5@5@+=feaafiart1ev1aaatCvAUfKttLearuWrP9MDH5MBPbIqV92AaeXatLxBI9gBaebbnrfifHhDYfgasaacH8akY=wiFfYdH8Gipec8Eeeu0xXdbba9frFj0=OqFfea0dXdd9vqai=hGuQ8kuc9pgc9s8qqaq=dirpe0xb9q8qiLsFr0=vr0=vr0dc8meaabaqaciaacaGaaeqabaqabeGadaaakeaaiiGacqWFdpWCdaqhaaWcbaGae8xTdugabaGaeGOmaidaaaaa@3137@ can not be derived in the same way. Instead of above likelihood function, we construct the following likelihood function by using joint conditional density of yi,

L(θ)=∑i=1nln⁡{E[f(yi|θ1,zi,wi)g(ζi|σζ2)]}     (17)
 MathType@MTEF@5@5@+=feaafiart1ev1aaatCvAUfKttLearuWrP9MDH5MBPbIqV92AaeXatLxBI9gBaebbnrfifHhDYfgasaacH8akY=wiFfYdH8Gipec8Eeeu0xXdbba9frFj0=OqFfea0dXdd9vqai=hGuQ8kuc9pgc9s8qqaq=dirpe0xb9q8qiLsFr0=vr0=vr0dc8meaabaqaciaacaGaaeqabaqabeGadaaakeaacqWGmbatcqGGOaakiiGacqWF4oqCcqGGPaqkcqGH9aqpdaaeWbqaaiGbcYgaSjabc6gaUnaacmqabaGaemyrauKaei4waSLaemOzayMaeiikaGIaemyEaK3aaSbaaSqaaiabdMgaPbqabaGccqGG8baFcqWF4oqCdaWgaaWcbaacbaGae4xmaedabeaakiabcYcaSiabdQha6naaBaaaleaacqWGPbqAaeqaaOGaeiilaWIaem4DaC3aaSbaaSqaaiabdMgaPbqabaGccqGGPaqkcqWGNbWzcqGGOaakcqWF2oGEdaWgaaWcbaGaemyAaKgabeaakiabcYha8jab=n8aZnaaDaaaleaacqWF2oGEaeaacqaIYaGmaaGccqGGPaqkcqGGDbqxaiaawUhacaGL9baaaSqaaiabdMgaPjabg2da9iabigdaXaqaaiabd6gaUbqdcqGHris5aaaa@6075@

Where *θ*_1 _= [*μ a d ζ*_*i *_σε2
 MathType@MTEF@5@5@+=feaafiart1ev1aaatCvAUfKttLearuWrP9MDH5MBPbIqV92AaeXatLxBI9gBaebbnrfifHhDYfgasaacH8akY=wiFfYdH8Gipec8Eeeu0xXdbba9frFj0=OqFfea0dXdd9vqai=hGuQ8kuc9pgc9s8qqaq=dirpe0xb9q8qiLsFr0=vr0=vr0dc8meaabaqaciaacaGaaeqabaqabeGadaaakeaaiiGacqWFdpWCdaqhaaWcbaGae8xTdugabaGaeGOmaidaaaaa@3137@]

f(yi|θ1,zi,wi)=12πσεexp⁡[−12σε2(yi−Mi−φiζi)T(yi−Mi−φiζi)]g(ζi|σζ2)=12πσζexp⁡(−ζi22σζ2)
 MathType@MTEF@5@5@+=feaafiart1ev1aaatCvAUfKttLearuWrP9MDH5MBPbIqV92AaeXatLxBI9gBaebbnrfifHhDYfgasaacH8akY=wiFfYdH8Gipec8Eeeu0xXdbba9frFj0=OqFfea0dXdd9vqai=hGuQ8kuc9pgc9s8qqaq=dirpe0xb9q8qiLsFr0=vr0=vr0dc8meaabaqaciaacaGaaeqabaqabeGadaaakeaafaqabeGabaaabaGaemOzayMaeiikaGIaemyEaK3aaSbaaSqaaiabdMgaPbqabaGccqGG8baFiiGacqWF4oqCdaWgaaWcbaGaeGymaedabeaakiabcYcaSiabdQha6naaBaaaleaacqWGPbqAaeqaaOGaeiilaWIaem4DaC3aaSbaaSqaaiabdMgaPbqabaGccqGGPaqkcqGH9aqpdaWcaaqaaiabigdaXaqaamaakaaabaGaeGOmaiJae8hWdahaleqaaOGae83Wdm3aaSbaaSqaaiab=v7aLbqabaaaaOGagiyzauMaeiiEaGNaeiiCaa3aamWaaeaacqGHsisldaWcaaqaaiabigdaXaqaaiabikdaYiab=n8aZnaaDaaaleaacqWF1oqzaeaacqaIYaGmaaaaaOGaeiikaGIaemyEaK3aaSbaaSqaaiabdMgaPbqabaGccqGHsislcqWGnbqtdaWgaaWcbaGaemyAaKgabeaakiabgkHiTiab=z8aMnaaBaaaleaacqWGPbqAaeqaaOGae8NTdO3aaSbaaSqaaiabdMgaPbqabaGccqGGPaqkdaahaaWcbeqaaiabdsfaubaakiabcIcaOiabdMha5naaBaaaleaacqWGPbqAaeqaaOGaeyOeI0Iaemyta00aaSbaaSqaaiabdMgaPbqabaGccqGHsislcqWFgpGzdaWgaaWcbaGaemyAaKgabeaakiab=z7a6naaBaaaleaacqWGPbqAaeqaaOGaeiykaKcacaGLBbGaayzxaaaabaGaem4zaCMaeiikaGIae8NTdO3aaSbaaSqaaiabdMgaPbqabaGccqGG8baFcqWFdpWCdaqhaaWcbaGae8NTdOhabaGaeGOmaidaaOGaeiykaKIaeyypa0ZaaSaaaeaacqaIXaqmaeaadaGcaaqaaiabikdaYiab=b8aWbWcbeaakiab=n8aZnaaBaaaleaacqWF2oGEaeqaaaaakiGbcwgaLjabcIha4jabcchaWnaabmaabaGaeyOeI0YaaSaaaeaacqWF2oGEdaqhaaWcbaGaemyAaKgabaGaeGOmaidaaaGcbaGaeGOmaiJae83Wdm3aa0baaSqaaiab=z7a6bqaaiabikdaYaaaaaaakiaawIcacaGLPaaaaaaaaa@9B93@

With derivative for *θ*_1_, we obtain

$$\left[\begin{array}{cccccc} \sum_{i=1}^{n}m_{i} & \sum_{i=1}^{n}m_{i}E(z_{i}) & \sum_{i=1}^{n}m_{i}E(w_{i}) & m_{1} & \cdots & m_{n}\\ \sum_{i=1}^{n}m_{i}E(z_{i}) & \sum_{i=1}^{n}m_{i}E(z_{i}^{2}) & \sum_{i=1}^{n}m_{i}E(z_{i}w_{i}) & m_{1}E(z_{1}) & \cdots & m_{n}E(z_{i})\\ \sum_{i=1}^{n}m_{i}E(w_{i}) & \sum_{i=1}^{n}m_{i}E(z_{i}w_{i}) & \sum_{i=1}^{n}m_{i}E(w_{i}^{2}) & m_{1}E(w_{1}) & \cdots & m_{n}E(w_{i})\\ m_{1} & m_{1}E(z_{1}) & m_{1}E(w_{1}) & m_{1}+\frac{\sigma_{\varepsilon }^{2}}{\sigma_{\zeta }^{2}} & \cdots & 0\\ \cdots & \cdots & \cdots & \cdots & \cdots & \cdots \\ m_{n} & m_{n}E(z_{i}) & m_{n}E(w_{i}) & 0 & \cdots & m_{n}+\frac{\sigma_{\varepsilon }^{2}}{\sigma_{\zeta }^{2}} \end{array}\right]\left[\begin{array}{c} \mu \\ a\\ d\\ \zeta_{1}\\ \cdots \\ \zeta_{n} \end{array}\right]=\left[\begin{array}{c} \sum_{i=1}^{n}m_{i}{\bar{y}}_{i}\\ \sum_{i=1}^{n}m_{i}E(z_{i}){\bar{y}}_{i}\\ \sum_{i=1}^{n}m_{i}E(w_{i}){\bar{y}}_{i}\\ m_{1}{\bar{y}}_{1}\\ \cdots \\ m_{n}{\bar{y}}_{n} \end{array}\right]\;\left(18\right)$$

and

σε2=1∑i=1nmi∑i=1nE[(yi−φiμ−ziφia−wiφid−φiζi)T(y˜i−φiμ−ziφia−wiφid−φiζi)]     (19)
 MathType@MTEF@5@5@+=feaafiart1ev1aaatCvAUfKttLearuWrP9MDH5MBPbIqV92AaeXatLxBI9gBaebbnrfifHhDYfgasaacH8akY=wiFfYdH8Gipec8Eeeu0xXdbba9frFj0=OqFfea0dXdd9vqai=hGuQ8kuc9pgc9s8qqaq=dirpe0xb9q8qiLsFr0=vr0=vr0dc8meaabaqaciaacaGaaeqabaqabeGadaaakeaaiiGacqWFdpWCdaqhaaWcbaGae8xTdugabaGaeGOmaidaaOGaeyypa0ZaaSaaaeaacqaIXaqmaeaadaaeWaqaaiabd2gaTnaaBaaaleaacqWGPbqAaeqaaaqaaiabdMgaPjabg2da9iabigdaXaqaaiabd6gaUbqdcqGHris5aaaakmaaqahabaGaemyrauKaei4waSLaeiikaGIaemyEaK3aaSbaaSqaaiabdMgaPbqabaGccqGHsislcqWFgpGzdaWgaaWcbaGaemyAaKgabeaakiab=X7aTjabgkHiTiabdQha6naaBaaaleaacqWGPbqAaeqaaOGae8NXdy2aaSbaaSqaaiabdMgaPbqabaGccqWGHbqycqGHsislcqWG3bWDdaWgaaWcbaGaemyAaKgabeaakiab=z8aMnaaBaaaleaacqWGPbqAaeqaaOGaemizaqMaeyOeI0Iae8NXdy2aaSbaaSqaaiabdMgaPbqabaGccqWF2oGEdaWgaaWcbaGaemyAaKgabeaakiabcMcaPmaaCaaaleqabaGaemivaqfaaOGaeiikaGIafmyEaKNbaGaadaWgaaWcbaGaemyAaKgabeaakiabgkHiTiab=z8aMnaaBaaaleaacqWGPbqAaeqaaOGae8hVd0MaeyOeI0IaemOEaO3aaSbaaSqaaiabdMgaPbqabaGccqWFgpGzdaWgaaWcbaGaemyAaKgabeaakiabdggaHjabgkHiTiabdEha3naaBaaaleaacqWGPbqAaeqaaOGae8NXdy2aaSbaaSqaaiabdMgaPbqabaGccqWGKbazcqGHsislcqWFgpGzdaWgaaWcbaGaemyAaKgabeaakiab=z7a6naaBaaaleaacqWGPbqAaeqaaOGaeiykaKIaeiyxa0faleaacqWGPbqAcqGH9aqpcqaIXaqmaeaacqWGUbGBa0GaeyyeIuoaaaa@8F4E@

Where

E[(yi−φiμ−ziφia−wiφid−φiζi)T(yi−φiμ−ziφia−wiφid−φiζi)]=(yi−φiμ)T(yi−φiμ)2+mia2E(zi2)+mid2E(wi2)−2(yi−φiμ)T[φiaE(zi)+φidE(wi)]+2miadE(ziwi)+miE(ζi2)     (20)
 MathType@MTEF@5@5@+=feaafiart1ev1aaatCvAUfKttLearuWrP9MDH5MBPbIqV92AaeXatLxBI9gBaebbnrfifHhDYfgasaacH8akY=wiFfYdH8Gipec8Eeeu0xXdbba9frFj0=OqFfea0dXdd9vqai=hGuQ8kuc9pgc9s8qqaq=dirpe0xb9q8qiLsFr0=vr0=vr0dc8meaabaqaciaacaGaaeqabaqabeGadaaakeaafaqaaeGabaaabaGaemyrauKaei4waSLaeiikaGIaemyEaK3aaSbaaSqaaiabdMgaPbqabaGccqGHsisliiGacqWFgpGzdaWgaaWcbaGaemyAaKgabeaakiab=X7aTjabgkHiTiabdQha6naaBaaaleaacqWGPbqAaeqaaOGae8NXdy2aaSbaaSqaaiabdMgaPbqabaGccqWGHbqycqGHsislcqWG3bWDdaWgaaWcbaGaemyAaKgabeaakiab=z8aMnaaBaaaleaacqWGPbqAaeqaaOGaemizaqMaeyOeI0Iae8NXdy2aaSbaaSqaaiabdMgaPbqabaGccqWF2oGEdaWgaaWcbaGaemyAaKgabeaakiabcMcaPmaaCaaaleqabaGaemivaqfaaOGaeiikaGIaemyEaK3aaSbaaSqaaiabdMgaPbqabaGccqGHsislcqWFgpGzdaWgaaWcbaGaemyAaKgabeaakiab=X7aTjabgkHiTiabdQha6naaBaaaleaacqWGPbqAaeqaaOGae8NXdy2aaSbaaSqaaiabdMgaPbqabaGccqWGHbqycqGHsislcqWG3bWDdaWgaaWcbaGaemyAaKgabeaakiab=z8aMnaaBaaaleaacqWGPbqAaeqaaOGaemizaqMaeyOeI0Iae8NXdy2aaSbaaSqaaiabdMgaPbqabaGccqWF2oGEdaWgaaWcbaGaemyAaKgabeaakiabcMcaPiabc2faDbqaceaa8gGaaCzcaiabg2da9iabcIcaOiabdMha5naaBaaaleaacqWGPbqAaeqaaOGaeyOeI0Iae8NXdy2aaSbaaSqaaiabdMgaPbqabaGccqWF8oqBcqGGPaqkdaahaaWcbeqaaiabdsfaubaakiabcIcaOiabdMha5naaBaaaleaacqWGPbqAaeqaaOGaeyOeI0Iae8NXdy2aaSbaaSqaaiabdMgaPbqabaGccqWF8oqBcqGGPaqkdaahaaWcbeqaaiabikdaYaaakiabgUcaRiabd2gaTnaaBaaaleaacqWGPbqAaeqaaOGaemyyae2aaWbaaSqabeaacqaIYaGmaaGccqWGfbqrcqGGOaakcqWG6bGEdaqhaaWcbaGaemyAaKgabaGaeGOmaidaaOGaeiykaKIaey4kaSIaemyBa02aaSbaaSqaaiabdMgaPbqabaGccqWGKbazdaahaaWcbeqaaiabikdaYaaakiabdweafjabcIcaOiabdEha3naaDaaaleaacqWGPbqAaeaacqaIYaGmaaGccqGGPaqkcqGHsislcqaIYaGmcqGGOaakcqWG5bqEdaWgaaWcbaGaemyAaKgabeaakiabgkHiTiab=z8aMnaaBaaaleaacqWGPbqAaeqaaOGae8hVd0MaeiykaKYaaWbaaSqabeaacqWGubavaaGccqGGBbWwcqWFgpGzdaWgaaWcbaGaemyAaKgabeaakiabdggaHjabdweafjabcIcaOiabdQha6naaBaaaleaacqWGPbqAaeqaaOGaeiykaKIaey4kaSIae8NXdy2aaSbaaSqaaiabdMgaPbqabaGccqWGKbazcqWGfbqrcqGGOaakcqWG3bWDdaWgaaWcbaGaemyAaKgabeaakiabcMcaPiabc2faDjabgUcaRiabikdaYiabd2gaTnaaBaaaleaacqWGPbqAaeqaaOGaemyyaeMaemizaqMaemyrauKaeiikaGIaemOEaO3aaSbaaSqaaiabdMgaPbqabaGccqWG3bWDdaWgaaWcbaGaemyAaKgabeaakiabcMcaPiabgUcaRiabd2gaTnaaBaaaleaacqWGPbqAaeqaaOGaemyrauKaeiikaGIae8NTdO3aa0baaSqaaiabdMgaPbqaaiabikdaYaaakiabcMcaPaaaaaa@EDA4@

so, we can simply utilize existing mixed model EM algorithm to find the MLE of parameters [9]. Followings are the EM steps for the mixed model analysis.

*Step 0*: Initialize all parameters with values in their legal domain, denoted by *θ*^(0)^.

*Step 1*: Compute the posterior probabilities of the three genotypes for each individual

$$\begin{array}{cc} p_{ik}^{*}=\frac{p_{ik}f(y_{i}|\theta_{1},z_{i},w_{i})}{\sum_{k=1}^{3}p_{ik}f(y_{i}|\theta_{1},z_{i}w_{i})} & \text{for }k=1,2,3 \end{array}\;\left(21\right)$$

*Step 2*: Compute all the expectations involved in the following maximization steps (same with the equation (8)).

*Step 3*: Find the posterior distribution of the random effect *p*_*i *_from equation (18). This posterior distribution turns out to be a mixture of three normal distributions with a mean

E(ζi)=σp2φV−1[yi−φμ−(pi1∗−pi3∗)φa−(pi2∗−pi1∗−pi3∗)φd]     (22)
 MathType@MTEF@5@5@+=feaafiart1ev1aaatCvAUfKttLearuWrP9MDH5MBPbIqV92AaeXatLxBI9gBaebbnrfifHhDYfgasaacH8akY=wiFfYdH8Gipec8Eeeu0xXdbba9frFj0=OqFfea0dXdd9vqai=hGuQ8kuc9pgc9s8qqaq=dirpe0xb9q8qiLsFr0=vr0=vr0dc8meaabaqaciaacaGaaeqabaqabeGadaaakeaacqWGfbqrcqGGOaakiiGacqWF2oGEdaWgaaWcbaGaemyAaKgabeaakiabcMcaPiabg2da9iab=n8aZnaaDaaaleaacqWGWbaCaeaacqaIYaGmaaGccqWFgpGzcqWGwbGvdaahaaWcbeqaaiabgkHiTiabigdaXaaakmaadmaabaGaemyEaK3aaSbaaSqaaiabdMgaPbqabaGccqGHsislcqWFgpGzcqWF8oqBcqGHsislcqGGOaakcqWGWbaCdaqhaaWcbaGaemyAaKMaeGymaedabaGaey4fIOcaaOGaeyOeI0IaemiCaa3aa0baaSqaaiabdMgaPjabiodaZaqaaiabgEHiQaaakiabcMcaPiab=z8aMjabdggaHjabgkHiTiabcIcaOiabdchaWnaaDaaaleaacqWGPbqAcqaIYaGmaeaacqGHxiIkaaGccqGHsislcqWGWbaCdaqhaaWcbaGaemyAaKMaeGymaedabaGaey4fIOcaaOGaeyOeI0IaemiCaa3aa0baaSqaaiabdMgaPjabiodaZaqaaiabgEHiQaaakiabcMcaPiab=z8aMjabdsgaKbGaay5waiaaw2faaaaa@6CBE@

and a variance

Var(ζi)=σζ2−φiV−1φiTσζ4     (23)
 MathType@MTEF@5@5@+=feaafiart1ev1aaatCvAUfKttLearuWrP9MDH5MBPbIqV92AaeXatLxBI9gBaebbnrfifHhDYfgasaacH8akY=wiFfYdH8Gipec8Eeeu0xXdbba9frFj0=OqFfea0dXdd9vqai=hGuQ8kuc9pgc9s8qqaq=dirpe0xb9q8qiLsFr0=vr0=vr0dc8meaabaqaciaacaGaaeqabaqabeGadaaakeaacqWGwbGvcqWGHbqycqWGYbGCcqGGOaakiiGacqWF2oGEdaWgaaWcbaGaemyAaKgabeaakiabcMcaPiabg2da9iab=n8aZnaaDaaaleaacqWF2oGEaeaacqaIYaGmaaGccqGHsislcqWFgpGzdaWgaaWcbaGaemyAaKgabeaakiabdAfawnaaCaaaleqabaGaeyOeI0IaeGymaedaaOGae8NXdy2aa0baaSqaaiabdMgaPbqaaiabdsfaubaakiab=n8aZnaaDaaaleaacqWF2oGEaeaacqaI0aanaaaaaa@4BD0@

*Step 4*: Update the population mean, additive effect and dominance effect by equation (16). The resulting equations are equivalent to equations (9) replacing *m*_*i *_with φTVi−1φ
 MathType@MTEF@5@5@+=feaafiart1ev1aaatCvAUfKttLearuWrP9MDH5MBPbIqV92AaeXatLxBI9gBaebbnrfifHhDYfgasaacPC6xNi=xI8qiVKYPFjYdHaVhbbf9v8qqaqFr0xc9vqFj0dXdbba91qpepeI8k8fiI+fsY=rqGqVepae9pg0db9vqaiVgFr0xfr=xfr=xc9adbaqaaeGacaGaaiaabeqaaeqabiWaaaGcbaGaeqOXdy2aa0baaSqaaaqaaiabdsfaubaakiabdAfawnaaDaaaleaacqWGPbqAaeaacqGHsislcqaIXaqmaaGccqaHgpGzdaWgaaWcbaaabeaaaaa@35CB@.

*Step 5*: Update the covariance matrix of the random effect

σζ2(1)=1n∑i=1nE(ζi2)=1n∑i=1n[Var(ζi)+E2(ζi)]     (24)
 MathType@MTEF@5@5@+=feaafiart1ev1aaatCvAUfKttLearuWrP9MDH5MBPbIqV92AaeXatLxBI9gBaebbnrfifHhDYfgasaacH8akY=wiFfYdH8Gipec8Eeeu0xXdbba9frFj0=OqFfea0dXdd9vqai=hGuQ8kuc9pgc9s8qqaq=dirpe0xb9q8qiLsFr0=vr0=vr0dc8meaabaqaciaacaGaaeqabaqabeGadaaakeaaiiGacqWFdpWCdaqhaaWcbaGae8NTdOhabaGaeGOmaiJaeiikaGIaeGymaeJaeiykaKcaaOGaeyypa0ZaaSaaaeaacqaIXaqmaeaacqWGUbGBaaWaaabmaeaacqWGfbqrcqGGOaakcqWF2oGEdaqhaaWcbaGaemyAaKgabaGaeGOmaidaaOGaeiykaKcaleaacqWGPbqAcqGH9aqpcqaIXaqmaeaacqWGUbGBa0GaeyyeIuoakiabg2da9maalaaabaGaeGymaedabaGaemOBa4gaamaaqadabaWaamWaaeaacqWGwbGvcqWGHbqycqWGYbGCcqGGOaakcqWF2oGEdaWgaaWcbaGaemyAaKgabeaakiabcMcaPiabgUcaRiabdweafnaaCaaaleqabaGaeGOmaidaaOGaeiikaGIae8NTdO3aaSbaaSqaaiabdMgaPbqabaGccqGGPaqkaiaawUfacaGLDbaaaSqaaiabdMgaPjabg2da9iabigdaXaqaaiabd6gaUbqdcqGHris5aaaa@623D@

*Step 6*: Update the residual variance by equation (19)

σ2=[∑i=1nmi]−1{∑i=1nmi[pi1∗(yi−μ−a+d−φiζi)T(yi−μ−a+d−φiζi)+     +pi2∗(y¯i−μ−d−φiζi)T(y¯i−μ−d−φiζi)     +pi3∗(y¯i−μ+a+d−φiζi)T(y¯i−μ+a+d−φiζi)]}
 MathType@MTEF@5@5@+=feaafiart1ev1aaatCvAUfKttLearuWrP9MDH5MBPbIqV92AaeXatLxBI9gBaebbnrfifHhDYfgasaacH8akY=wiFfYdH8Gipec8Eeeu0xXdbba9frFj0=OqFfea0dXdd9vqai=hGuQ8kuc9pgc9s8qqaq=dirpe0xb9q8qiLsFr0=vr0=vr0dc8meaabaqaciaacaGaaeqabaqabeGadaaakeGacaa4baaIcuaabmqadeaaaeaaiiGacqWFdpWCdaahaaWcbeqaaiabikdaYaaakiabg2da9maadmaabaWaaabCaeaacqWGTbqBdaWgaaWcbaGaemyAaKgabeaaaeaacqWGPbqAcqGH9aqpcqaIXaqmaeaacqWGUbGBa0GaeyyeIuoaaOGaay5waiaaw2faamaaCaaaleqabaGaeyOeI0IaeGymaedaaOWaaiqabeaadaaeWbqaaiabd2gaTnaaBaaaleaacqWGPbqAaeqaaOWaamqaaeaacqWGWbaCdaqhaaWcbaGaemyAaKMaeGymaedabaGaey4fIOcaaOGaeiikaGIaemyEaK3aaSbaaSqaaiabdMgaPbqabaGccqGHsislcqWF8oqBcqGHsislcqWGHbqycqGHRaWkcqWGKbazcqGHsislcqWFgpGzdaWgaaWcbaGaemyAaKgabeaakiab=z7a6naaBaaaleaacqWGPbqAaeqaaOGaeiykaKYaaWbaaSqabeaacqWGubavaaGccqGGOaakcqWG5bqEdaWgaaWcbaGaemyAaKgabeaakiabgkHiTiab=X7aTjabgkHiTiabdggaHjabgUcaRiabdsgaKjabgkHiTiab=z8aMnaaBaaaleaacqWGPbqAaeqaaOGae8NTdO3aaSbaaSqaaiabdMgaPbqabaGccqGGPaqkcqGHRaWkaiaawUfaaaWcbaGaemyAaKMaeyypa0JaeGymaedabaGaemOBa4ganiabggHiLdaakiaawUhaaaqaciaazbaanOGaaCzcaiaaxMaacqGHRaWkcqWGWbaCdaqhaaWcbaGaemyAaKMaeGOmaidabaGaey4fIOcaaOGaeiikaGIafmyEaKNbaebadaWgaaWcbaGaemyAaKgabeaakiabgkHiTiabeY7aTjabgkHiTiabdsgaKjabgkHiTiab=z8aMnaaBaaaleaacqWGPbqAaeqaaOGae8NTdO3aaSbaaSqaaiabdMgaPbqabaGccqGGPaqkdaahaaWcbeqaaiabdsfaubaakiabcIcaOiqbdMha5zaaraWaaSbaaSqaaiabdMgaPbqabaGccqGHsislcqWF8oqBcqGHsislcqWGKbazcqGHsislcqWFgpGzdaWgaaWcbaGaemyAaKgabeaakiab=z7a6naaBaaaleaacqWGPbqAaeqaaOGaeiykaKcabiqbaKcaaeGcaKHcauIcaeCdcaWLjaGaaCzcaiaaxMaacaWLjaGaey4kaSYaaiGabeaadaWacaqaaiabdchaWnaaDaaaleaacqWGPbqAcqaIZaWmaeaacqGHxiIkaaGccqGGOaakcuWG5bqEgaqeamaaBaaaleaacqWGPbqAaeqaaOGaeyOeI0Iae8hVd0Maey4kaSIaemyyaeMaey4kaSIaemizaqMaeyOeI0Iae8NXdy2aaSbaaSqaaiabdMgaPbqabaGccqWF2oGEdaWgaaWcbaGaemyAaKgabeaakiabcMcaPmaaCaaaleqabaGaemivaqfaaOGaeiikaGIafmyEaKNbaebadaWgaaWcbaGaemyAaKgabeaakiabgkHiTiab=X7aTjabgUcaRiabdggaHjabgUcaRiabdsgaKjabgkHiTiab=z8aMnaaBaaaleaacqWGPbqAaeqaaOGae8NTdO3aaSbaaSqaaiabdMgaPbqabaGccqGGPaqkaiaaw2faaaGaayzFaaaaaaaa@D8C2@

*Step7*: Repeat from step 1 to step 6 until a certain convergence criterion is reached.

MLE of parameters in both model (2) and (10) are iteratively solved at specific location on chromosomes using EM algorithm and the QTL position and effects are determined by means of likelihood ratio statistics in chromosome or genome scanning.

## Simulation studies

A series of simulation experiments were used to compare the efficiency and behaviour of two mapping methods based on the repeatability model with simple analysis using the mean phenotype for a trait with repeat records. We simulated a single chromosome of 100 cM long with 11 evenly spaced codominant markers for an F2 population with sample size *n *= 100 and a single QTL was put at position 25 cM (between markers 3 and 4). Under the null model, the QTL was assigned a value of zero for both the additive and dominance effects. The empirical critical values of likelihood ratio statistics for testing the presence of the QTL were obtained by simulating 1000 replicates. Under the alternative model, nonzero and equal additive and dominance effects were simulated. The simulations were replicated 100 times. Empirical power was calculated by counting the number of runs in which test statistics were greater than the critical values.

Factor considered include the QTL size, measured as the proportion of the phenotypic variance explained by the QTL (also called the QTL heritability), the number of replicates and σζ2
 MathType@MTEF@5@5@+=feaafiart1ev1aaatCvAUfKttLearuWrP9MDH5MBPbIqV92AaeXatLxBI9gBaebbnrfifHhDYfgasaacH8akY=wiFfYdH8Gipec8Eeeu0xXdbba9frFj0=OqFfea0dXdd9vqai=hGuQ8kuc9pgc9s8qqaq=dirpe0xb9q8qiLsFr0=vr0=vr0dc8meaabaqaciaacaGaaeqabaqabeGadaaakeaaiiGacqWFdpWCdaqhaaWcbaGae8NTdOhabaGaeGOmaidaaaaa@314D@:σε2
 MathType@MTEF@5@5@+=feaafiart1ev1aaatCvAUfKttLearuWrP9MDH5MBPbIqV92AaeXatLxBI9gBaebbnrfifHhDYfgasaacH8akY=wiFfYdH8Gipec8Eeeu0xXdbba9frFj0=OqFfea0dXdd9vqai=hGuQ8kuc9pgc9s8qqaq=dirpe0xb9q8qiLsFr0=vr0=vr0dc8meaabaqaciaacaGaaeqabaqabeGadaaakeaaiiGacqWFdpWCdaqhaaWcbaGae8xTdugabaGaeGOmaidaaaaa@3137@ i.e the variance ratio of permanent environmental effect to random environmental effect. The QTL size was set at three levels: *a *= *d *= 0.265, 0.577, 0.943 correspond to the three levels of *h*^2 ^= 0.05, 0.10, 0.20 respectively. The number of replicates was examined at five levels: *m *= 1, 3, 5, 10, 15, and σζ2
 MathType@MTEF@5@5@+=feaafiart1ev1aaatCvAUfKttLearuWrP9MDH5MBPbIqV92AaeXatLxBI9gBaebbnrfifHhDYfgasaacH8akY=wiFfYdH8Gipec8Eeeu0xXdbba9frFj0=OqFfea0dXdd9vqai=hGuQ8kuc9pgc9s8qqaq=dirpe0xb9q8qiLsFr0=vr0=vr0dc8meaabaqaciaacaGaaeqabaqabeGadaaakeaaiiGacqWFdpWCdaqhaaWcbaGae8NTdOhabaGaeGOmaidaaaaa@314D@: σε2
 MathType@MTEF@5@5@+=feaafiart1ev1aaatCvAUfKttLearuWrP9MDH5MBPbIqV92AaeXatLxBI9gBaebbnrfifHhDYfgasaacH8akY=wiFfYdH8Gipec8Eeeu0xXdbba9frFj0=OqFfea0dXdd9vqai=hGuQ8kuc9pgc9s8qqaq=dirpe0xb9q8qiLsFr0=vr0=vr0dc8meaabaqaciaacaGaaeqabaqabeGadaaakeaaiiGacqWFdpWCdaqhaaWcbaGae8xTdugabaGaeGOmaidaaaaa@3137@ = 1:4, 2:3, 2.5:2.5, 3:2, 4:1, remaining σζ2
 MathType@MTEF@5@5@+=feaafiart1ev1aaatCvAUfKttLearuWrP9MDH5MBPbIqV92AaeXatLxBI9gBaebbnrfifHhDYfgasaacH8akY=wiFfYdH8Gipec8Eeeu0xXdbba9frFj0=OqFfea0dXdd9vqai=hGuQ8kuc9pgc9s8qqaq=dirpe0xb9q8qiLsFr0=vr0=vr0dc8meaabaqaciaacaGaaeqabaqabeGadaaakeaaiiGacqWFdpWCdaqhaaWcbaGae8NTdOhabaGaeGOmaidaaaaa@314D@ + σε2
 MathType@MTEF@5@5@+=feaafiart1ev1aaatCvAUfKttLearuWrP9MDH5MBPbIqV92AaeXatLxBI9gBaebbnrfifHhDYfgasaacH8akY=wiFfYdH8Gipec8Eeeu0xXdbba9frFj0=OqFfea0dXdd9vqai=hGuQ8kuc9pgc9s8qqaq=dirpe0xb9q8qiLsFr0=vr0=vr0dc8meaabaqaciaacaGaaeqabaqabeGadaaakeaaiiGacqWFdpWCdaqhaaWcbaGae8xTdugabaGaeGOmaidaaaaa@3137@ = 5.0.

The *j*th phenotypic value of individual *i *was simulated by using the repeatability model:

*y*_*ij *_= *μ *+ *z*_*i *_*a *+ *w*_*i *_*d *+ *ξ*_*i*_*σ*_*ζ *_+ *η*_*ij*_*σ*_*ε *_     (25)

Where both *ξ*_*i *_and *η *_*ij *_are the random numbers from standard normal distribution. 

The results of all simulations consistently show that under the same experimental condition, (1) using the repeatability model can significantly increase the statistical power of QTL detecting compared with simple analysis using the mean phenotype, (2) the position and effects of QTL, especially the proportion of phenotypic variance contributed by QTL were more accuracy estimated by using the repeatability model than using the genetic mapping model without permanent environmental effects to analyze mean phenotype. The superiority of the repeatability model over the simple analysis using the mean phenotype performs in evidence under the condition of the low QTL heritability.

The effects of number of replications on the efficiency and behaviour of the two methods were investigated only at variance ratio of permanent environmental effect to random environmental effect of 1:1. The results of simulations were listed in Table 1 and 2, respectively, by different mapping method. Notices that the simulated results at *m *= 1 (no replication) only correspond to the mapping method based on the mean phenotype for no solution by using the repeatability model. As expected, the statistical power of QTL detecting with replication is higher than no replication, based on either the mean phenotype or the repeatability model. The estimation of QTL parameters show a general tendency to improve as the number of replications increases.

**Table 1:** Effects of the number of replications on the mapping analysis based on the repeatability model

			Estimate							
			
*a *and *d*	*h*^2^	Replicate	Power	Position	*a*	*d*	*h*^2^	σp2 MathType@MTEF@5@5@+=feaafiart1ev1aaatCvAUfKttLearuWrP9MDH5MBPbIqV92AaeXatLxBI9gBaebbnrfifHhDYfgasaacH8akY=wiFfYdH8Gipec8Eeeu0xXdbba9frFj0=OqFfea0dXdd9vqai=hGuQ8kuc9pgc9s8qqaq=dirpe0xb9q8qiLsFr0=vr0=vr0dc8meaabaqaciaacaGaaeqabaqabeGadaaakeaaiiGacqWFdpWCdaqhaaWcbaGaemiCaahabaGaeGOmaidaaaaa@30FE@	σε2 MathType@MTEF@5@5@+=feaafiart1ev1aaatCvAUfKttLearuWrP9MDH5MBPbIqV92AaeXatLxBI9gBaebbnrfifHhDYfgasaacH8akY=wiFfYdH8Gipec8Eeeu0xXdbba9frFj0=OqFfea0dXdd9vqai=hGuQ8kuc9pgc9s8qqaq=dirpe0xb9q8qiLsFr0=vr0=vr0dc8meaabaqaciaacaGaaeqabaqabeGadaaakeaaiiGacqWFdpWCdaqhaaWcbaGae8xTdugabaGaeGOmaidaaaaa@3137@	*LOD*
0.4189	0.05	3	37	28.65(1.946)	0.615(0.061)	0.594(0.028)	0.116(0.007)	2.183(0.069)	2.544(0.046)	15.31(0.810)
		5	46	27.63(1.506)	0.626(0.291)	0.545(0.226)	0.108(0.041)	2.224(0.050)	2.555(0.018)	15.39(0.493)
		10	63	26.55(0.923)	0.698(0.224)	0.661(0.178)	0.134(0.042)	2.375(0.037)	2.548(0.014)	18.85(0.535)
		15	86	26.71(1.441)	0.496(0.028)	0.544(0.190)	0.093(0.003)	2.294(0.035)	2.532(0.010)	14.85(0.386)
0.6086	0.10	3	80	25.98(0.635)	0.724(0.036)	0.670(0.025)	0.142(0.006)	2.239(0.049)	2.605(0.030)	18.18(0.635)
		5	83	26.55(0.922)	0.699(0.022)	0.661(0.018)	0.134(0.042)	2.375(0.034)	2.548(0.014)	18.85(0.535)
		10	87	26.43(0.640)	0.650(0.024)	0.668(0.017)	0.130(0.004)	2.310(0.029)	2.547(0.010)	20.09(0.538)
		15	93	25.78(0.678)	0.637(0.232)	0.632(0.138)	0.119(0.037)	2.412(0.028)	2.537(0.078)	18.77(0.515)
0.9129	0.20	3	99	24.04(0.507)	0.937(0.038)	0.960(0.023)	0.223(0.007)	2.322(0.051)	2.643(0.028)	29.25(0.928)
		5	99	24.89(0.319)	0.881(0.027)	0.922(0.015)	0.207(0.005)	2.374(0.024)	2.636(0.013)	29.12(0.640)
		10	100	24.90(0.330)	0.949(0.025)	0.917(0.157)	0.213(0.005)	2.397(0.027)	2.573(0.010)	32.86(0.655)
		15	100	25.16(0.359)	0.914(0.241)	0.884(0.014)	0.120(0.005)	2.473(0.030)	2.569(0.008)	31.26(0.651)

*h*^2 ^is the proportion of phenotypic variance explained by the QTL. The variance ratio of permanent environmental effect to random environmental effect is fixed as 1:1. Standard deviations are in parentheses.

**Table 2:** Effects of the number of replications on the simple analysis using the mean phenotype

			Estimate						
			
*a *and *d*	*h*^2^	Replicate	Power	Position	*a*	*d*	*h*^2^	*σ*^*2*^	*LOD*
0.4189	0.05	1	21	38.36(5.188)	0.673(0.101)	0.505(0.097)	0.096(0.004)	4.266(0.139)	17.00(0.812)
		3	34	26.65(0.888)	0.559(0.317)	0.560(0.207)	0.197(0.007)	2.189 (0.034)	15.39(0.493)
		5	42	29.50(1.026)	0.653(0.294)	0.541(0.296)	0.178(0.059)	2.743(0.512)	17.34(0.529)
		10	56	27.04(0.954)	0.719(0.241)	0.694(0.180)	0.218(0.061)	2.911(0.337)	20.92(0.559)
		15	81	26.16(1.521)	0.496(0.029)	0.560(0.021)	0.173(0.005)	2.469(0.038)	16.87(0.416)
0.6086	0.10	1	57	23.89(1.774)	0.767(0.050)	0.777(0.040)	0.120(0.039)	4.785(0.082)	17.22(0.606)
		3	78	25.39(0.660)	0.661(0.024)	0.639(0.016)	0.234(0.063)	2.256(0.027)	23.31(0.531)
		5	81	27.04(0.954)	0.719(0.241)	0.694(0.018)	0.219(0.061)	2.911(0.034)	20.92(0.559)
		10	84	26.23(0.602)	0.667(0.245)	0.683(0.167)	0.223(0.065)	2.600(0.279)	21.65(0.564)
		15	87	25.79(0.672)	0.652(0.233)	0.647(0.147)	0.211(0.060)	2.586(0.026)	20.77(0.529)
0.9129	0.20	1	97	25.21(0.563)	1.003(0.043)	0.970(0.030)	0.208(0.005)	4.800(0.082)	23.44(0.725)
		3	100	25.10(0.302)	0.909(0.233)	0.916(0.015)	0.357(0.007)	2.311(0.025)	38.04(0.773)
		5	99	25.00(0.305)	0.886(0.027)	0.930(0.016)	0.306(0.007)	2.974(0.033)	30.93(0.653)
		10	100	25.08(0.307)	0.952(0.026)	0.932(0.016)	0.335(0.007)	2.689(0.027)	34.94(0.673)
		15	99	25.07(0.288)	0.929(0.025)	0.914(0.015)	0.330(0.007)	2.659(0.026)	33.65(0.678)

*h*^2 ^is the proportion of phenotypic variance explained by the QTL. The variance ratio of permanent environmental effect to random environmental effect is fixed as 1:1. Standard deviations are in parentheses.

We have also investigated the impact of the variance ratio of permanent environmental effect to random environmental effect on differences in mapping performance between the two methods. The results of simulations fixing five replications were listed in Table 3. The difference in variance between permanent environmental effect and random environmental effect is greater under fixing total variance of random effects, the superiority of the mapping method based on the repeatability model over the mean phenotype is clearer in the statistical power of QTL detecting. The possible reasons are that either the large variance of random environmental effect made reliability of the individual's mean phenotype value low or the variance of residual error in model (2) increases with the variance of permanent environmental effect increased.

**Table 3:** Comparisons of the mapping analysis based on the repeatability model with the simple analysis using the mean phenotype under the conditions of different the variance ratios of permanent environmental effects to random environmental effects

			Estimate							
			
h^2^	σp2 MathType@MTEF@5@5@+=feaafiart1ev1aaatCvAUfKttLearuWrP9MDH5MBPbIqV92AaeXatLxBI9gBaebbnrfifHhDYfgasaacH8akY=wiFfYdH8Gipec8Eeeu0xXdbba9frFj0=OqFfea0dXdd9vqai=hGuQ8kuc9pgc9s8qqaq=dirpe0xb9q8qiLsFr0=vr0=vr0dc8meaabaqaciaacaGaaeqabaqabeGadaaakeaaiiGacqWFdpWCdaqhaaWcbaGaemiCaahabaGaeGOmaidaaaaa@30FE@:σε2 MathType@MTEF@5@5@+=feaafiart1ev1aaatCvAUfKttLearuWrP9MDH5MBPbIqV92AaeXatLxBI9gBaebbnrfifHhDYfgasaacH8akY=wiFfYdH8Gipec8Eeeu0xXdbba9frFj0=OqFfea0dXdd9vqai=hGuQ8kuc9pgc9s8qqaq=dirpe0xb9q8qiLsFr0=vr0=vr0dc8meaabaqaciaacaGaaeqabaqabeGadaaakeaaiiGacqWFdpWCdaqhaaWcbaGae8xTdugabaGaeGOmaidaaaaa@3137@	Method	Power	Position	*a*	*d*	*h*^2^	σp2 MathType@MTEF@5@5@+=feaafiart1ev1aaatCvAUfKttLearuWrP9MDH5MBPbIqV92AaeXatLxBI9gBaebbnrfifHhDYfgasaacH8akY=wiFfYdH8Gipec8Eeeu0xXdbba9frFj0=OqFfea0dXdd9vqai=hGuQ8kuc9pgc9s8qqaq=dirpe0xb9q8qiLsFr0=vr0=vr0dc8meaabaqaciaacaGaaeqabaqabeGadaaakeaaiiGacqWFdpWCdaqhaaWcbaGaemiCaahabaGaeGOmaidaaaaa@30FE@	σε2 MathType@MTEF@5@5@+=feaafiart1ev1aaatCvAUfKttLearuWrP9MDH5MBPbIqV92AaeXatLxBI9gBaebbnrfifHhDYfgasaacH8akY=wiFfYdH8Gipec8Eeeu0xXdbba9frFj0=OqFfea0dXdd9vqai=hGuQ8kuc9pgc9s8qqaq=dirpe0xb9q8qiLsFr0=vr0=vr0dc8meaabaqaciaacaGaaeqabaqabeGadaaakeaaiiGacqWFdpWCdaqhaaWcbaGae8xTdugabaGaeGOmaidaaaaa@3137@ or *σ*^*2 *^	*LOD*
0.05	1: 4	Repeat	72	25.59(0.919)	0.471(0.021)	0.494(0.013)	0.076(0.003)	0.889(0.022)	4.080(0.025)	17.27(0.484)
		Mean	60	25.52(0.870)	0.491(0.024)	0.507(0.015)	0.199(0.006)		1.711(0.234)	19.68(0.525)
	2: 3	Repeat	44	27.59(1.662)	0.544(0.032)	0.540(0.022)	0.098(0.006)	1.776(0.039)	3.023(0.024)	15.79(0.495)
		Mean	42	26.47(1.415)	0.549(0.030)	0.527(0.027)	0.177(0.006)		2.418(0.039)	17.39(0.500)
	3: 2	Repeat	38	24.97(1.441)	0.607(0.034)	0.576(0.023)	0.110(0.004)	2.745(0.060)	2.048(0.018)	14.54(0.389)
		Mean	37	25.76(1.456)	0.601(0.034)	0.585(0.026)	0.162(0.006)		3.158(0.054)	16.16(0.452)
	4: 1	Repeat	33	30.57(2.141)	0.668(0.050)	0.558(0.035)	0.128(0.063)	3.604(0.067)	1.007(0.010)	14.04(0.437)
		Mean	26	30.62(2.321)	0.717(0.051)	0.598(0.043)	0.171(0.007)		3.694(0.071)	16.74(0.570)
0.10	1: 4	Repeat	97	25.01(0.408)	0.643(0.019)	0.622(0.013)	0.115(0.036)	0.917(0.023)	4.042(0.019)	25.05(0.586)
		Mean	94	24.93(0.411)	0.648(0.020)	0.628(0.013)	0.267(0.007)		1.765(0.023)	26.56(0.600)
	2: 3	Repeat	86	26.32(0.815)	0.667(0.022)	0.635(0.014)	0.122(0.033)	1.890(0.030)	3.085(0.015)	19.28(0.440)
		Mean	84	26.34(0.827)	0.669(0.023)	0.643(0.014)	0.215(0.054)		2.518(0.029)	20.80(0.459)
	3: 2	Repeat	83	25.53(0.612)	0.655(0.028)	0.679(0.017)	0.137(0.004)	2.718(0.035)	2.079(0.013)	17.76(0.422)
		Mean	83	25.73(0.750)	0.659(0.029)	0.689(0.018)	0.199(0.006)		3.145(0.033)	19.37(0.451)
	4: 1	Repeat	64	25.14(1.043)	0.703(0.029)	0.686(0.018)	0.143(0.004)	3.812(0.051)	1.007(0.007)	16.27(0.430)
		Mean	61	25.44(0.997)	0.725(0.032)	0.751(0.022)	0.192(0.006)		3.898(0.051)	18.32(0.437)

*h*^2 ^is the proportion of phenotypic variance explained by the QTL. There are 100 individuals each with five records. Standard deviations are in parentheses.

## Discussion

For a trait with repeat records, we proposed use of the repeatability model to map QTL, which distinguishes from simple analysis using the mean phenotype not only in the data analyzed but essentially in the model adopted. Simple analysis using the mean phenotype was based on regular genetic model for mapping QTL in linecross, which excluded the permanent environmental effects. The excluded permanent environmental effects were deposited to the residual error, decreasing the accuracy of estimation for QTL parameters, which was strictly proved in the relevant books to statistic models [e.g., [10,11]]. Of course, the loss of data information has also influenced the performance of mapping QTL based on the mean phenotype.

Replication required either the experimental conditions must be the same when multiple records were observed only from one individual or the genetic backgrounds must be the identical for each individual while those records were from multiple individuals. If the former was not satisfied, then such "repeat" records observed became longitudinal data, such as test-day records of milk production and body weight in cattle, were genetically analysed using the random regresion model which is essentially the repeatability model nested submodels of time [12-14]. Besides cloned individuals and progencies from each plant in RIL, the later was hard to be satisfied. For example, there were incompletely same genetic backgrounds among individuals within a family and F3 progenies from one F2 individual. To improve the efficiency of detecting QTL using such data, the genetic backgrounds should be at least taken into account in the analysis [7], furthermore, the repeatability model may be a good choice for directly analyzing such "repeat" records.

Although we demonstrate the statistical method of QTL mapping using a F_2 _population as an example, other more simple or complex designs, such as backcross population and full-sib family can also be extended. Assuming only one QTL in the model considered here is to conveniently investigate efficiency of presented method based on various estimates. If a trait is controlled by multiple loci, the composite interval mapping [15,16] or Bayesian mapping [e.g., [17,18]] will be proposed for mapping those QTLs by incorporating marker-cofactors outside the scanning interval or all the QTLs into the model (9).

## Authors' contributions

RQY coordinated the study, developed the foundational principle of the method and wrote the computing program and the paper. FM was responsible for the simulation experiment and carried out the analysis of results.
